# The Nordic model fails to protect vulnerable children

**DOI:** 10.1016/j.lanepe.2025.101217

**Published:** 2025-01-15

**Authors:** Věra Skalická, Terje Andreas Eikemo

**Affiliations:** a Department of Psychology, Norwegian University of Science and Technology, Trondheim, Norway; b Centre for Global Health Inequalities Research (CHAIN), Department of Sociology and Political Science, Norwegian University of Science and Technology (NTNU), Trondheim, Norway

Over 69 million children live in poverty in the world's richest countries^1^ despite the UN Convention on the Rights of the Child guaranteeing every child's right to survival and comprehensive development.

Recent research highlights the causal link between low income and adverse health outcomes in children and adolescents.^2^ Childhood poverty can lead to poor physical and mental health, which in turn can diminish socioeconomic attainment in young adulthood, reinforcing a cycle of disadvantage that persists into later life.^3^^,^^4^ This underscores the need for early public health policies focused on aiding children in poverty.

Policymakers often look towards the Nordic countries for solutions, as they are considered model countries for tackling poverty through their generous, universal welfare programs and high living standards. However, the Nordic countries are not immune to this issue, with approximately 1 in 10 children in Denmark, Finland, Norway, and Iceland living in poverty, with Sweden having the highest rate of 18%.^1^ Furthermore, Norway ranks 35 out of 39 OECD and EU countries in its efforts to reduce child poverty between 2012 and 2021.^1^

UNICEF Norway has criticized the Norwegian government for failing to take adequate measures to support children and families in need.^5^ According to Statistics Norway, 10.6% of children in Norway (around 102,600 children) lived in families with household income below 60 per cent of the median income from 2020 to 2022.^6^ Although this is a slight decrease compared to the years 2019–2021 (11.3%), the number was only 3.3% for the period 1999–2001.^6^

Effective policy implementations are needed to address income inequalities, which have been largely neglected in Norway’s welfare system over recent decades. Specifically, recommendations include substantial increases in child benefit, and taxing it, to ensure that the benefit is universal, and helps the poorest families the most.^7^ The rise of low-income families, particularly single-parent households, underlines this necessity.

Despite Norway's reputation for promoting equality and equal opportunities, the reality shows an alarming increase in income disparities, intensified since the early 2000s by tax policies favoring wealthier individuals.^8^^,^^9^ Housing price surges have led young adults to rely on parental financial assistance for purchasing homes, fostering a cycle of intergenerational socioeconomic disadvantage.

To combat poverty effectively, policymakers must consider the multiple pathways that lead to it. Alongside fiscal measures targeting income and housing, interventions must address the emotional and resource stressors that poverty instigates in families. Research shows that low parental income translates to poor child health through increased parenting stress and limited financial or time resources to invest in children’s development.^7^ Addressing these pathways necessitates universal public health initiatives, including school-based interventions, as well as targeted parenting support for vulnerable populations.

Direct financial policies are paramount, including providing subsidies for early childhood care and afterschool programs. In parallel, there is a pressing need to raise the standards of care in early childhood education and the broader education system. Universal measures, such as the introduction of free school lunches and extended physical education hours, would not only aid the general pupil population but would particularly benefit children from economically disadvantaged families unable to afford meals or recreational activities.

Enhancing the understanding of social determinants impacting children's health and mental well-being is crucial. Effective interventions should be informed by strong causal inference methods utilizing longitudinal data from representational samples across diverse social settings and countries.

A commitment to cross-national collaboration in research initiatives, particularly the Growing Up in Digital Europe (GUIDE) Study, is vital.^10^ This extensive longitudinal study will track the well-being of children and young people across Europe over the next 34 years. Investments in cross-border data infrastructures are critical to tracking and responding to the evolving needs of children in poverty.

The realities of child poverty in wealthy nations, including Nordic states, present an urgent call for robust social policies. Such policies must be designed with a scale and intensity that corresponds with the level of disadvantage.

Addressing child poverty is not merely a matter of ensuring equitable opportunities but is also fundamentally linked to public health outcomes. A comprehensive strategy that combines increased financial assistance with focused intervention programs is essential for meaningful progress. Equally important is the involvement of children and adolescents in these initiatives, allowing their voices to influence the collective efforts to alleviate family poverty.

Enhanced child benefits and investments in cross-national research infrastructures are crucial to break the cycle of disadvantage, promote health equity, and secure a brighter future for children across socioeconomic divides.

## Contributors

VS and TAE both contributed equally to the conceptualisation of the study and writing of original draft and editing.

## Declaration of interests

We declare no competing interests.
